# Occupational accidents in Russia and the Russian Arctic

**DOI:** 10.3402/ijch.v72i0.20458

**Published:** 2013-03-19

**Authors:** Alexey A. Dudarev, Igor P. Karnachev, Jon Øyvind Odland

**Affiliations:** 1 Hygiene Department, Northwest Public Health Research Centre, St. Petersburg, Russia; 2 Kola Research Laboratory for Occupational Health, Kirovsk, Murmansk Oblast, Russia; 3 AMAP Secretariat, University of Tromsø, Tromsø, Norway

**Keywords:** occupational accidents, injuries, fatal accidents, occupational safety, labour conditions, Russian Arctic

## Abstract

**Background:**

According to official statistics, the rate of occupational accidents (OAs) and fatal injuries in Russia decreased about 5-fold and 2-fold, respectively, from 1975 to 2010, but working conditions during this period had the opposite trend; for example, the number of people who work in unfavourable and hazardous conditions (particularly since 1991) has increased significantly.

**Methods:**

This review summarises the results of a search of the relevant peer-reviewed literature published in Russia and official statistics on OAs and occupational safety in Russia and the Russian Arctic in 1980–2010.

**Results:**

The occupational safety system in Russia has severely deteriorated in the last 2 decades, with legislators tending to promote the interests of industry and business, resulting in the neglect of occupational safety and violation of workers’ rights. The majority of workers are employed in conditions that do not meet rules of safety and hygiene. More than 60% of OAs can be attributed to management practices – violation of safety regulations, poor organisation of work, deficiency of certified occupational safety specialists and inadequate personnel training. Research aimed at improving occupational safety and health is underfunded. There is evidence of widespread under-reporting of OAs, including fatal accidents. Three federal agencies are responsible for OAs recording; their data differ from each other as they use different methodologies. The rate of fatal OAs in Russia was 3–6 times higher than in Scandinavian countries and about 2 times higher compared to United States and Canada in 2001. In some Russian Arctic regions OAs levels are much higher.

**Conclusions:**

Urgent improvement of occupational health and safety across Russia, especially in the Arctic regions, is needed.

In tsarist Russia, regular reviews of occupational accidents (OAs) and assessment of industrial hazards began after the introduction of the *Notice of Occupational Accidents* in 1903 and the *Law on Workers Insurance* in 1912. According to this law, the health insurance funds of factories and plants came under the supervision of All-Russian Insurance Board, Provincial Insurance Public Office, Police, and Department of Public Security and Factory Inspection, which strengthened considerably the regulation of relationships between factory owners and workers. In 1910 the 11th Pirogov's Congress in Saint Petersburg introduced a special card for the uniform recording of injuries in Russia and put in place the collection of injuries statistics. The aim of the card was to record data on OAs at factories, mines and railways.

When the Bolsheviks came to power, the People's Commissariat introduced in 1921 the obligatory investigation of all OAs by technical and sanitary inspectors at enterprises. Since 1922, all enterprises were required to register all OAs and to send a notice (in a prescribed form) to the local labour inspector, which in effect was the real beginning of collection and processing of statistical data on OAs in Russia. In 1925, a list of especially hazardous works prohibited for women was adopted, followed by the standard scheme and procedure of registration, reporting and investigation of OAs in 1927. The introduction of occupational insurance, which followed the appearance of trade unions and insurance companies, made a significant contribution to the development of the methods of risk identification and analysis, and the prevention of OAs and illnesses. In 1927, a nationwide network of occupational safety departments at enterprises was established with a complete staff of specialised engineers and hygienists.

## Occupational safety in Russia today

Nowadays, Russian laws and regulations exist to address occupational safety, but unfortunately they are more a declaration of intent and are routinely ignored by employers. OAs investigation is carried out in accordance with *Regulations of investigation and registration of occupational accidents*, approved by the Russian Government in 1999, the 2002 resolution of the Ministry of Labour on *Approval of documents required for investigation and registration of occupational accidents* and regulations on *Details of the occupational accidents investigation in specific industries and institutions*. Formally, the law protects the employees. The employer is held responsible for the OA. The injured person must be paid temporary disability benefits comparable to the average wage from the company's funds. In case of permanent disability resulting from injury or other damage to health, the person must be awarded a life pension. Moreover, the compensation of material damage to the injured person for the disability must be equal to the difference between the average monthly wages lost and disability pension. In practice the law does not work well and the employees are often unprotected.

The occupational safety system in Russia has severely deteriorated in the past 2 decades. The mortality rate of working-age people from “external” causes (accidents, poisonings and injuries) now corresponds to the rate in Russia a century ago, and is much higher than in other developed countries.

High rate of OAs is caused first of all by poor working conditions. Official data of the Federal State Statistics Service (Rosstat) clearly show that the highest proportion of workers employed in conditions that do not meet safety and hygienic standards can be found in Arctic, Siberian and Far East regions, namely Murmansk region, Komi Republic, Kemerovo region, Chukotka, Kamchatka and Koryakia.

According to the Rosstat, every year more than 60% of the OAs are related to poor management – failure to meet safety standards, poor organisation of work, deficiency of occupational safety certified specialists, lack of personnel training and periodic medical examinations of workers and non-compliance with labour regulations, and so on. The number of fatal OAs has significantly increased during the past years. During the period 1991–2009, the average rate of fatalities in the Russian coal-mining industry was 54±12 per 100 million tonnes of coal; it is 15 times higher than in the United States (3.46±0.55). The increase in frequency and scale of coal-mining accidents (e.g. the Raspadskaya mine accident in 2010 which killed about 100 miners) against a background of decreasing productivity is the direct result of negligence of safety rules by employers (1).

The Ministry of Labour which existed in the former USSR and then the Russian Federation was abolished in 2004, and its functions were transferred to the Ministry of Health and Social Development. After the transfer, occupational safety in Russia suffered severe decline. The official position was that there were no reasons for concern, as the rate of OAs are decreasing. Governmental policy appears to have drastically changed the occupational safety to the worse (2).

In Russia at the federal level there is no single organisation responsible for labour protection and the development of state policies on the management of worker safety. There is no funding for research studies aimed at increasing occupational safety and health (3, 4). The availability of powerless and low-paid migrants and illegal immigrants easily solves the problem of labour protection in many enterprises and there is little incentive to improve conditions (5). The amount of money spent, not the beneficial health outcomes, is the sole criterion of activity, and funds earmarked for labour protection often end up in the coffers of some privileged groups (6).

The training programme for occupational health inspectors is rudimentary: for example, 0.5–2 hours suffice for the studying of the principles of injury prevention, and a similar duration is enough for studying the investigation and reporting of OAs; less than 1 hour would suffice to study ventilation, lighting, noise and vibration. Modern methods in the analysis of accidents are not taught at all. There is no quality control of such programmes rather than the lists of enrolled “listeners”. The number of training centres grows every year, and the number of “trained” inspectors is estimated to be in the millions. Again, some privileged groups benefit from such activities (5, 6).

## Official statistics on occupational accidents: 1980–2010

Official statistical data on OAs in the former USSR and Russia from 1980–2010 were obtained from the Russian Statistical Yearbooks (7–9). Data for 7 northern and far eastern regions (mainly during the 2000–2008 period) were from the regional Statistical Yearbooks of Arkhangelsk Oblast, Murmansk Oblast, Karelia republic, Komi republic, Chukotka Autonomous Okrug, Kamchatka Oblast and Magadan Oblast (10–16).

Data on OAs in different industrial sectors in Russia are given in Table I. According to Rosstat, the highest OA rates are observed in manufacturing, agriculture, hunting and forestry. Between 1975 and 2010 the rate of OAs in Russia decreased about 5-fold and fatal injuries halved. Yet, working conditions during this period had the opposite trend: the number of people who work in unfavourable and hazardous conditions (particularly since 1991) has increased significantly.

**Table I:** Number of occupational accidents in different industries, 2004–2010, Russia

Types of industry							

	Total	Agriculture, hunting, forestry	Mining	Manufacturing	Production and distribution of electric energy, gas and water	Construction	Transport and communication
Thousands people							
*Total number of accidents*							
2004	87.8	19.9	5.5	32.3	3.3	7.1	8.5
2005	77.7	15.9	4.9	28.9	3.1	7.2	7.9
2006	70.7	12.9	4.2	27.1	3.0	6.6	7.4
2007	66.1	10.6	3.9	26.6	2.6	6.6	7.2
2008	58.3	7.9	3.3	23.8	2.4	6.3	6.6
2009	46.1	6.7	2.7	17.0	2.1	4.9	5.6
2010	47.7	6.1	2.8	18.7	2.2	4.6	5.9
*Number of fatal accidents*							
2004	3.3	0.7	0.3	0.8	0.2	0.5	0.4
2005	3.1	0.6	0.3	0.8	0.2	0.5	0.4
2006	2.9	0.5	0.3	0.7	0.2	0.5	0.4
2007	3.0	0.5	0.4	0.7	0.2	0.6	0.4
2008	2.6	0.4	0.2	0.6	0.2	0.6	0.3
2009	2.0	0.3	0.2	0.4	0.2	0.5	0.3
2010	2.0	0.3	0.3	0.5	0.2	0.4	0.3
Per 1,000 workers							
*Total number of accidents*							
2004	3.4	5.8	5.1	3.9	1.9	4.4	2.4
2005	3.1	5.3	4.7	3.6	1.7	4.4	2.2
2006	2.9	4.9	4.0	3.5	1.7	4.1	2.0
2007	2.7	4.5	3.7	3.4	1.4	3.8	2.0
2008	2.5	3.9	3.3	3.2	1.3	3.6	1.9
2009	2.1	3.6	2.8	2.5	1.2	3.1	1.7
2010	2.2	3.6	3.0	2.9	1.3	3.0	1.8
*Number of fatal accidents*							
2004	0.129	0.213	0.318	0.092	0.107	0.333	0.114
2005	0.124	0.198	0.279	0.096	0.107	0.312	0.112
2006	0.119	0.206	0.271	0.087	0.096	0.332	0.099
2007	0.124	0.215	0.389	0.085	0.116	0.346	0.099
2008	0.109	0.184	0.213	0.080	0.100	0.327	0.099
2009	0.090	0.173	0.191	0.065	0.086	0.284	0.076
2010	0.094	0.172	0.274	0.072	0.089	0.234	0.086

**Fig. 1:**
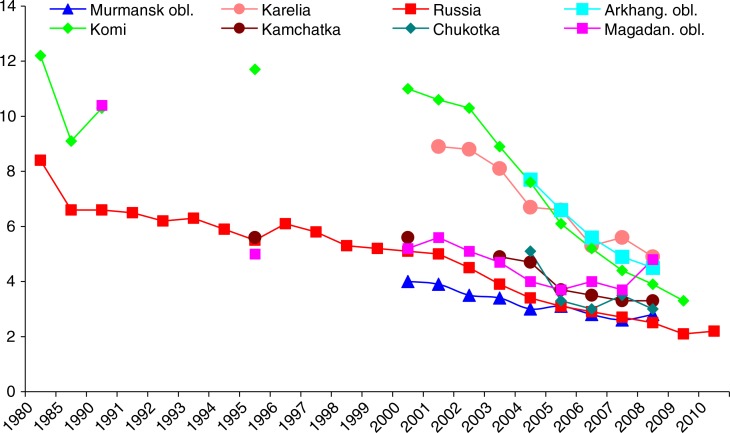
Occupational accidents in Russian Arctic regions compared to Russia (1980–2009), per 1,000 workers.

Figure 1 shows the rate of OAs in Arctic regions compared to Russia. They all show the decreasing trend. The rates for Murmansk, Magadan, Chukotka and Kamchatka are very similar to the Russian one, whereas Arkhangelsk, Karelia and Komi (and also the western European regions of Russia) have higher rates, and a more steep decline.

**Fig. 2:**
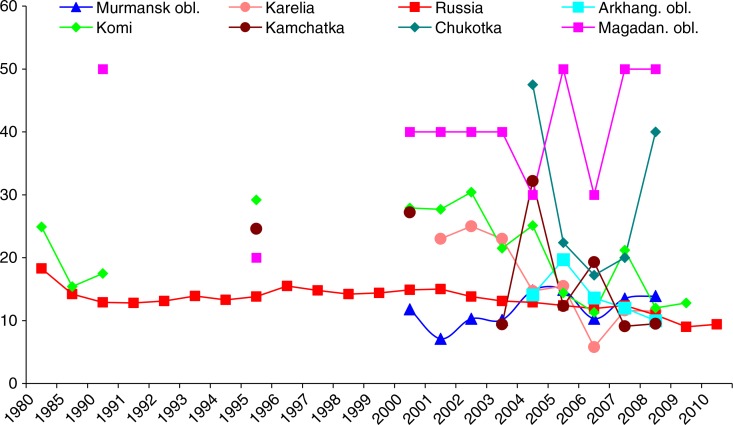
Fatal occupational accidents in Russian Arctic regions compared to Russia (1980–2009), per 100,000 workers.

In terms of fatal OAs, the Russian rate during the 1980–2010 period was within the range of 10–20 cases/100,000 workers. Due to the smaller number of cases, the rates for the Arctic regions fluctuate more widely, especially in Magadan, Chukotka and Kamchatka. Karelia and Komi demonstrate similar trends with a tendency to decrease (Fig. 2).

## Comparison with circumpolar countries

Comparison of indicators of occupational injuries in the Russian Federation with other countries is difficult due to the different approaches to data collection. Many observers are of the opinion that the OA rates in Russia should be much higher than published official statistics. The International Labour Organisation (ILO) subscribes to this point of view also, based on comparison of OA data of a number of European countries. With some 64 million workers in the Russian economy (in 2003) the number of accidents appear lower than in Germany or Great Britain which have half the number of employed. Internationally, one in 500–2,000 cases of OAs (resulting in disability lasting more than 3 days) are fatal. In Germany and Finland the ratio is about 1:1,000–1:1,200. If these ratios were applied to Russia, one would expect at least 3.5 to 5 million non-fatal cases. Yet, according to Rosstat the total number of OAs in 2003 was 107,000, and in 2004, less than 100,000. It is possible that the true number of OAs are some 30–50 times higher than reported (4).

Even taking under-reporting into consideration, the rate of fatal OAs in Russia is still higher than the Scandinavian countries, Canada and the United States (Table II), according to an international comparative study providing global estimates on OAs (17).

**Table II:** Occupational fatal accidents for insured/covered people in circumpolar countries in 2001, per 100,000 employed

Rate of occupational fatal accidents	
Finland	2.9
Sweden	1.9
Norway	3.2
Denmark	3.4
Iceland	1.7
Canada	6.4
USA	5.2
Russia	11.0

## System of registration and reporting of occupational accidents in Russia

In Russia some officials have the tendency to consider information on labour conditions as state and commercial secret. The Federal State Statistics Service's OA reporting form emphasises the confidential nature of the information it contains. There is also a requirement to define the degree of liability of the insured person in percentage terms. There is much in the practice of investigation, processing, recording and classification of OAs in Russia that are open to criticism.

During the Soviet period, the total number of OAs in the former Russian Soviet Federative Socialist Repblic (RSFSR) was about 600–800,000 per year during the 1970s and 1980s. In the 1990s, with the “democratisation”, liberalisation and market capitalisation of the economy, the number of OAs dramatically fell, reaching about 100,000 in 2003–2004. Part of this decrease was due to a drop in production by about 40% in the country during that period but the main reason for such dramatic “decline” of OAs was direct concealment. Large-scale concealment of OAs is an open secret but appropriate steps are not taken. Methods of OAs concealment include “custom-made” reports of forensic medical experts, for example death from electrocution or heat stress misattributed to a heart attack. Coercing the family of the victim into agreeing not to perform an official investigation, destruction of material evidence, bribery and blackmail of eyewitnesses, concealment of true information on working conditions (state of buildings, constructions, equipment and means of protection) are also common (2, 5).

According to the current legislation, investigation of OAs is carried out by the commission formed by the employer, who is clearly in a conflict of interest position. Labour inspectors are obliged to investigate only “cases with serious consequences”, usually no more than 20% of OAs. With about one labour inspector per 1,000 employers in the Russian Federation in 2006, most employers would not be inspected more often than once in 10 years. The productivity and quality of the inspectors is low (2). In Russia there is no practical means to ensure compliance with labour legislation by employers. Under such circumstances, it is difficult to have confidence in official statistics on OAs, including fatal ones (4, 18).

In the Russian Federation 3 state organisations are responsible for the recording and analysis of OAs: Federal Inspection of Labour (Rostrud), Federal State Statistics Service (Rosstat) and Federal Social Insurance Fund (FSS). OAs statistical data reported by these federal organisations differ from each other, because they use different methodologies. Rostrud deals with all OAs, Rosstat keeps records of OAs in a limited number of economic sectors using sample surveys, covering about 25–30% of the workforce, and FSS takes into account only OAs that have been officially recognised as insurable events. Given these different data sources for OA, which is more accurate?; and on which should management decisions regarding occupational safety be based? Rostrud data is considered more reliable for 3 reasons: (1) experts in this department record the maximum number of OAs in the industrial sector; (2) labour inspectors have no reason (for the department's benefit) to understate deliberately the OAs statistics; and (3) the department monitors and supervises directly the OA investigation. Labour inspectors annually reveal about 3,000 concealed OAs, of which approximately 250–300 are fatal (18).

Other Federal agencies contribute their own particular “adjustment” to OA (including fatal) statistics. The Russian State Fire Control Service investigates fire accidents independently. A fire in a workplace may or may not be deemed an OA, and some deaths from fires are not reported as fatal OAs. The Russian State Traffic Safety Inspectorate investigating traffic accidents does not differentiate occupational and non-occupational accidents. The Ministry of Health Care and Social Development does not include fatal outcomes of occupational diseases in its official statistics, even though the health care system generally reports fatal outcomes from common diseases (4).

As an example of such statistical inconsistency we can look at official OAs data in Murmansk Oblast in 2008. According to the Murmansk Oblast Ministry of Social Development the total number of occupational injured persons was 125, including 30 deaths and 44 seriously injured (19). According to the Murmansk Oblast Statistical Yearbook (16) the total number of injured persons at work was 495, including 25 deaths.

Results of workplace certification in Murmansk Oblast in 2009 (with 17,000 workers, among them 10,500 women) revealed that only 32% of workplaces could be considered “optimal and acceptable” labour conditions, and 68% of workplaces as hazardous and harmful (20).

## Conclusions

To determine the precise number of occupational injuries and deaths it is necessary to establish an efficient mechanism of inter-agency interaction and collaboration, standardisation of primary data recording and reporting protocols, and implementation of a verification procedure. Health care institutions, through the Ministry of Public Health, must notify the Federal Inspection of Labour and other relevant agencies about all cases. Regional and National OAs registers should be established. As we have stated repeatedly in our companion papers, radical improvement of occupational safety in Russia is urgently needed.
